# Editorial

**Published:** 1841-02-06

**Authors:** 


### THE MEDICAL EXAMINER.

PHILADELPHIA, FEBRUARY 6, 1841.

### MEDICAL EVIDENCE.

The disagreement of doctors has been a standing theme for the jokes of the facetious. In essentials, generally more apparent than real, it is nevertheless at times thrust before the public in a way to impress unfavourably even the most reflecting among lookers on. Physicians of talent and repute are seen to differ upon points of legal medicine, involving life or character. The intricacy of the particular question mooted, the meagreness of the facts bearing upon it, are not allowed for, and the public mind passes to a general belief in the uncertainty and worthlessness of medical testimony. From time to time, too, there spring up a series of criminal cases, puzzling in their nature, and depending for solution upon the testimony of physicians, which may either be conflicting in character, or run counter to the current of popular prejudice. Within a short time several cases of this description have occurred. The opposing evidence of the distinguished chemists, Orfila and Beral, at the trial of Madame Laffarge, in France—the testimony in a recent suspected case of poisoning at Boston—and in one or two criminal cases in this city, involving the subject of insanity, have severally afforded plausible grounds for sneers at the discrepancy and fallacy of medical evidence. The ingenuity of advocates, too, has not been wanting, in turning to account and amplifying apparent variances in the opinions of medical witnesses. It is no difficult task for a clever lawyer to draw on a nervous physician, unaccustomed to the quirks and quibbles of courts, from statement to statement to conclusions, the exact bearing of which he does not at the moment perceive, but which, reüttered with slight variation of language, produce impressions neither authorized nor intended by the witness. Unimportant differences, too, on speculative points, are readily caught at to play off one physician against another and weaken the value of the evidence which is really germaine to the matter. An impression is thus fastened upon the public mind, inimical to the due administration of justice, and hurtful to the cause of science and of truth.

A law journal in Boston, commenting on a late criminal trial in that city, hints that whenever physicians figure in the witness box, there are as many opinions as witnesses. This is very unjust. On the broad questions of medical jurisprudence there is quite as much agreement of opinion as upon other scientific subjects. In most points of toxicology, of medical and surgical pathology, there will be found great unanimity in the testimony of well educated physicians. Occasionally will start up vexed questions which admit of differences of opinion—but these are quite rare. They mostly refer to the delicate subject of insanity, in which the opinion of the medical jurist is, we think, of least value, and where doubtful points are better finally referred to the common sense of mankind. With an exact knowledge of the facts, any intelligent man can decide upon the sanity and responsibility of an individual; nor is it surprising that physicians, like other men, differ in cases of intricacy. A very small portion of the 'glorious uncertainty of the law' can with justice be charged upon medical witnesses.

### METEOROLOGICAL REGISTER—BILLS OF MORTALITY.

We commence to-day the publication of a series of meteorological observations from the beginning of the current year, together with the bills of mortality for the city and liberties of Philadelphia—to be continued hereafter weekly. The former will be found full and valuable; the latter are somewhat loose and inaccurate, but contain much interesting statistical matter.

